# Antileptospiral activity of xanthones from *Garcinia mangostana* and synergy of gamma-mangostin with penicillin G

**DOI:** 10.1186/1472-6882-13-182

**Published:** 2013-07-19

**Authors:** Weeranuch Seesom, Amornmart Jaratrungtawee, Sunit Suksamrarn, Chantana Mekseepralard, Piniti Ratananukul, Wasana Sukhumsirichart

**Affiliations:** 1 Department of Biochemistry, Faculty of Medicine, Srinakharinwirot University, Sukhumvit 23, Bangkok 10110, Thailand; 2 Department of Chemistry and Center of Excellence for Innovation in Chemistry, Faculty of Science, Srinakharinwirot University, Sukhumvit 23, Bangkok 10110, Thailand; 3 Department of Microbiology, Faculty of Medicine, Srinakharinwirot University, Sukhumvit 23, Bangkok 10110, Thailand; 4 Office of Higher Education Commission, Ministry of Education, Bangkok 10400, Thailand

**Keywords:** Leptospira, Mangosteen, Xanthones, Gamma-Mangostin, Synergy, Penicillin G

## Abstract

**Background:**

Leptospirosis, one of the most widespread zoonotic infectious diseases worldwide, is caused by spirochetes bacteria of the genus *Leptospira*. The present study examined inhibitory activity of purified xanthones and crude extracts from *Garcinia mangostana* against both non-pathogenic and pathogenic leptospira. Synergy between γ-mangostin and penicillin G against leptospires was also determined.

**Methods:**

Minimal inhibitory concentrations (MIC) of crude extracts and purified xanthones from *G*. *mangostana* and penicillin G for a non-pathogenic (*L*. *biflexa* serovar Patoc) and pathogenic (*L*. *interrogans* serovar Bataviae, Autumnalis, Javanica and Saigon) leptospires were determined by using broth microdilution method and alamar blue. The synergy was evaluated by calculating the fractional inhibitory concentration (FIC) index.

**Results:**

The results of broth microdilution test demonstrated that the crude extract and purified xanthones from mangosteen possessed antileptospiral activities. The crude extracts were active against all five serovars of test leptospira with MICs ranging from 200 to ≥ 800 μg/ml. Among the crude extracts and purified xanthones, garcinone C was the most active compound against both of pathogenic (MIC =100 μg/ml) and non-pathogenic leptospira (MIC = 200 μg/ml). However, these MIC values were higher than those of traditional antibiotics. Combinations of γ-mangostin with penicillin G generated synergistic effect against *L*. *interrogans* serovars Bataviae, Autumnalis and Javanica (FIC = 0.52, 0.50, and 0.04, respectively) and no interaction against *L*. *biflexa* serovar Patoc (FIC =0.75). However, antagonistic activity (FIC = 4.03) was observed in *L*. *interrogans* serovar Saigon.

**Conclusions:**

Crude extracts and purified xanthones from fruit pericarp of *G*. *mangostana* with significant antibacterial activity may be used to control leptospirosis. The combination of xanthone with antibiotic enhances the antileptospiral efficacy.

## Background

Leptospirosis is an important infectious disease widespread worldwide. This disease is associated with illness or death in humans and causes economic loss in animals [1]. The agent that causes leptospirosis is spirochetes bacteria of the genus *Leptospira*, which includes pathogenic species (*L*. *interrogans*) and non-pathogenic species (*L*. *biflexa*). The pathogenic species can infect both of human and animals and widely distributed in the environment [2,3]. Outbreaks normally occur during the rainy season, coinciding with flooded areas [4]. Leptospires appear in the blood during the first 7–10 days after infection, after that the organism can be found in fresh urine [5]. Leptospirosis in humans has traditionally been treated with antibiotics such as penicillin G [6-9], doxycycline, cefotaxime, ceftriaxone, azithromycin, erythromycin, and ampicillin. The investigation of 24 antimicrobials for growth inhibition of 26 *Leptospira* spp. serovars was determined using a broth microdilution technique which was simple, fast, and reliable and it was found that some antimicrobials showed excellent *in vitro* activity against *Leptospira* spp. [10].

Apart from antibiotics, several bacteria, viruses, and fungi have been reported to be sensitive to xanthones which are secondary metabolites found in some higher plant families, fungi, and lichens [11,12]. They have been classified into five groups: simple oxygenated xanthones, xanthone glycosides, prenylated xanthones, xanthonolignoids, and miscellaneous xanthones [13,14]. The prenylated xanthones are isolated from pericarp, whole fruit, bark, and leaves of mangosteen which is a tropical tree cultivated in tropical rainforest of some Southeast Asia countries such as Indonesia, Malaysia, and Thailand. To date, over sixty-eight xanthones have been identified in the mangosteen fruit [15]. The xanthones obtained from the mangosteen fruit give remarkable biological activities such as α-, β-, and γ-mangostins, garcinone E, 8-desoxygartanin, and gartanin [16]. The garcinone B, α-, and β-mangostins exhibited the most potent inhibitory effect against *Mycobacterium tuberculosis*[17]. The α-mangostin has been reported to exhibit antifungal and antiviral activities [18]. Several xanthones from pericarp of mangosteen are used as medicinal agents for the treatment of skin infections, wounds [19], and diarrhea [20]. The mangosteen pericarp extracts were also found to have a high antioxidant activity which reduced the reactive oxygen species (ROS) [21]. The α- and γ-mangostins isolated from the fruit wall of *G*. *mangostana* are bioactive substances containing anti-inflammatory [22-24], anti-cancer [25-27] and anti-malarial [28] activities. In addition, xanthones from mangosteen have inhibitory effects on the growth of HIV [29], *Candida albicans*[30], *Staphylococcus aureus*[31], *Pseudomonas aeruginosa*, *Salmonella typhimurium*, and *Bacillus subtilis*[32], and anti-acne bacteria [33].

Combinations of antibiotics or plant extracts have been used in medicine to broaden the antimicrobial spectrum and to generate synergistic effects [34]. For example, the combination of plant extracts and antibiotics against *S*. *aureus* isolated from clinical specimens [35] and synergism between antipsychotic agents, prochlorperazine and methdilazine against bacteria [36]. As xanthones have been reported to demonstrate many antimicrobial effects, and penicillin G is an antibiotics traditionally used to treat leptospirosis in humans, this study was therefore designed to investigate the antimicrobial activities of four crude extracts and five xanthones isolated from pericarp of *G*. *mangostana*, and synergistic effect between a xanthone and penicillin G against *Leptospira* spp.

## Methods

### Leptospira isolates and cultured condition

A non-pathogenic *L*. *biflexa* serovar Patoc (serogroup Semaranga) and four pathogenic *L*. *interrogans* serovar Bataviae (serogroup Bataviae), Autumnalis (serogroup Autumnalis), Saigon (serogroup Louisiana) and Javanica (serogroup Javanica) were obtained from the Department of Medical Sciences, Ministry of Public Health, Nonthaburi, Thailand. The leptospires were grown in Ellinghausen, McCullough, Johnson, and Harris (EMJH) medium (Difco™, USA) at 30°C for 7 days.

### Mangosteen and xanthones isolation

**Figure 1:**
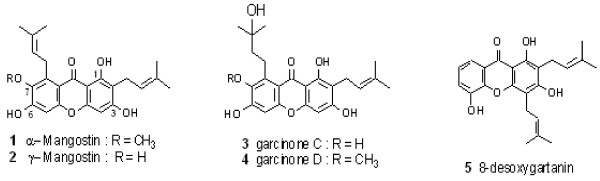
**Structure of xanthones isolated from the fruit of *G*. *mangostana *pericarp.** 1. α-mangostin, 2. γ-mangostin, 3. garcinone C, 4. garcinone D, and 5. 8-desoxygartanin.

The fruit of mangosteen was collected from Kombang District, Chantaburi Province, Thailand in 2007. A voucher specimen (Porntip Wongnapa No. 002) is deposited at the Faculty of Science, Ramkhamhaeng University, Thailand. Four crude extracts and five prenylated xanthones as shown in Table 1 were isolated from the fruit mangosteen as follows. Powdered of fruit pericarp (100 g) was extracted using ethyl acetate and followed by ethanol for 48 h each by using a Soxhlet apparatus. After the solvent was removed under reduced pressure, the crude extracts SS-WS01 (9 g, yellow solid) and SS-WS02 (8 g, dark brown solid) were obtained. Crude extract SS-WS03 (8 g, brown solid) was yielded from another 100 g-portion of the pericarp powder in a similar way but employing ethanol as extraction solvent. The extract SS-WS04 (9 g, dark red solid) was also prepared in a likewise manner as for SS-WS03 but using methanol in place of ethanol. Five major prenylated xanthones including α-mangostin (1), γ-mangostin (2), garcinone C (3), garcinone D (4), and 8-desoxygartanin (5) (Figure 1 and Table 1) were purified from the fruit pericarp and identified by using NMR and MS analysis as previously described [32]. The purity of these xanthones exceeded 95%, as determined by LC analysis [37]. The crude extracts and purified xanthones (dried-form) were dissolved in absolute dimethyl sulfoxide (DMSO) (Merck, Germany) to a concentration of 8 mg/ml and used as stock solution. The working solution was prepared by diluting the stock solution with EMJH medium to a concentration of 800 μg/ml.

**Table 1:** **Minimal inhibitory concentrations (MIC) of four crude extracts and five xanthones purified from*G*.*mangostana*against one non-pathogenic and four pathogenic leptospira**

**Compounds**				**MIC (μg/ml)**				
**Code**	**MW**	**Structure**	**Type**	**Patoc**	**Bataviae**	**Autumnalis**	**Javanica**	**Saigon**
SS-WS01	-	ND	Crude extract	≥ 800	≥800	400	400	≥800
SS-WS02	-	ND	Crude extract	≥800	400	200	400	≥800
SS-WS03	-	ND	Crude extract	≥800	≥800	400	200	≥800
SS-WS04	-	ND	Crude extract	≥800	400	200	200	400
**1**	410	C_24_H2_6_O_6_	α-Mangostin	≥800	400	≥800	100	100
**2**	396	C_23_H_24_O_6_	γ-Mangostin	200	100	≥800	100	100
**3**	414	C_23_H_26_O_7_	Garcinone C	200	100	100	100	100
**4**	428	C_24_H_28_O_7_	Garcinone D	≥800	≥800	≥800	200	200
**5**	380	C_23_H_24_O_5_	8-Desoxygartanin	≥800	≥800	400	400	200
*Penicillin G				6.25	1.56	3.13	0.39	0.78

*ND* Not determine; Positive control, EMJH medium containing 10% DMSO with leptospires; Negative control, EMJH medium containing 10% DMSO.

### Preparation of antibiotic

Stock solution of pencillin G (1 mg/ml) was prepared by dissolving 1 mg reagent grade of penicillin G powder (Amresco, USA) in 1 ml sterile-distilled water. The working solution (100 μg/ml) was prepared by diluting the stock solution with sterile-distilled water.

### Bacterial susceptibility testing

Antileptospiral assay were carried out using broth microdilution test [38,39]. Active leptospiral cultures were prepared in EMJH medium and grown at 30°C for 7 days. For assay, density of leptospires was determined by using PENTA SQUARE® plastic counting chamber (Vacutest Kima, Italy) under dark-field microscope. The culture was then diluted in EMJH medium to reach a bacterial density of 2 × 10^6^ cells/ml [38].

Two fold serial dilution of the test crude extracts or xanthones at concentration ranging from 50 to 800 μg/ml were prepared in EMJH medium containing 10% DMSO in a sterile 96-well round bottomed plate, final volume of 100 μl per well. A 100 μl volume of leptospira suspension (2 × 10^6^ cells/ml) was added to each well. Each plate included positive controls (EMJH containing 10% DMSO and leptospires without xanthones) and negative control (EMJH containing 10% DMSO) [39]. The plate was mixed and incubated at 30°C for 7 days. Then, each well was added with 20 μl of 10× alamar blue which is an oxidant-reduction indicator that changes colour from dark blue to bright pink in response to chemical reduction of the growth medium in the presence of bacterial viability. The plate was further incubated at 30°C for 1 day. The bacterial growth was observed by colour changing of the indicator and confirmed by measuring absorbance at 570 nm and 600 nm using ELISA reader. The MIC was defined as the lowest concentration of the crude extracts or xanthones that exhibited complete inhibition of microbial growth. The MIC of penicillin G was also performed as mentioned above, but test concentrations ranged from 0.025 to 50 μg/ml. All tests were carried out in duplicate.

### Determination of MIC of combined γ-mangostin and penicillin G

Fifty microliters of two-fold serial dilution of penicillin G (final concentration ranging from 0.0125 to 6.25 μg/ml) was pipetted into well containing 50 μl of γ-mangostin (final concentration ranging from 1.56 to 50 μg/ml). After mixing, 100 μl of leptospira inoculum (2 × 10^6^ cells/ml) was added to each well. The plate was performed in the same conditions used to determine the MIC of the crude extracts and xanthones. The MIC of combination was deemed to be the lowest concentration of both γ-mangostin and penicillin G which inhibited the growth of leptospires in the same well.

### Evaluation of the synergistic effect

Synergy was evaluated by calculating the fractional inhibitory concentration (FIC) index as described previously [36]: FIC index = FIC_A_ + FIC_B_ = [A]/MIC_A_ + [B]/MIC_B_, where [A] and [B] were the concentrations of penicillin G and γ-mangostin in combination, respectively. MIC_A_ and MIC_B_ were the MIC of penicillin G and γ-mangostin, respectively. Synergy testing was conducted according to guidelines established by the American Society for Microbiology, Instruction to Authors (1995) [40]. The FIC index was interpreted as follows: synergy, <0.5; partial synergy, 0.5-0.75; additive effect, 0.76-1.0; indifference, >1.0-4.0; and antagonism, >4.0.

## Results

### MIC of crude extracts and purified xanthones

Four crude extracts and five purified xanthones purified from pericarp of mangosteen were evaluated for antimicrobial activity against non-pathogenic and pathogenic leptospira. All four crude extracts were active against all serovars of test pathogenic leptospira with MICs ranging from 200 to ≥ 800 μg/ml whereas they had low activity for non-pathogenic leptospira, *L*. *biflexa* serovar Patoc with the MIC value of greater than or equal to 800 μg/ml (Table 1). The antileptospiral activity of five purified xanthones was variable in the ranged of 100 to ≥ 800 μg/ml with garcinone C demonstrating the highest activity (MICs ranging from 100 to 200 μg/ml) for both non-pathogenic and pathogenic leptospira.

### Synergy of γ-mangostin with penicillin G

All test leptospira were susceptible to penicillin G with different susceptibility between *L*. *biflexa* serovar Patoc (MIC 6.25 μg/ml) and *L*. *interrogans* including serovars Bataviae, Autumnalis, Javanica and Saigon (MICs 0.39 to 3.13 μg/ml) (Table 2). γ-Mangostin was found to have high antibacterial activity (MICs ranged from 100 to 200 μg/ml) against both non-pathogenic and pathogenic leptospira, except for *L*. *interrogans* serovars Autumnalis (MIC ≥ 800 μg/ml). The combination of penicillin G and γ-mangostin showed lower MICs of both compounds, apart from penicillin G when tested against *L*. *interrogans* serovar Saigon, gave higher MIC (3.13 μg/ml). This result indicated an increase in antileptospiral activity. The calculated FIC index demonstrated synergy for *L*. *interrogans* serovar Javanica, Autumnalis, and Bataviae (FIC = 0.04, 0.50, and 0.52, respectively). However, no interaction (FIC = 0.75) and antagonistic activity (FIC = 4.03) were shown against *L*. *biflexa* serovar Patoc and *L*. *interrogans* serovar Saigon, respectively (Table 2).

**Table 2:** **Susceptibility of*Leptospira*serovars to penicillin G, γ-Mangostin and the combination of both compounds**

**Leptospiral serovar**	**MIC (μg/ml)**				FIC* index	**Antileptospiral effect**
	**Before combination**		**After combination**			
	**Penicillin G**	**γ-Mangostin**	**Penicillin G**	**γ-Mangostin**		
Patoc	6.25	200	3.13	50	0.75	No interaction
Autumnalis	3.13	≥800	1.56	≤1.56	0.50	Synergy
Bataviae	1.56	100	0.78	≤1.56	0.52	Synergy
Javanica	0.39	100	≤0.01	≤1.56	0.04	Synergy
Saigon	0.78	100	3.13	≤1.56	4.03	Antagonism

*FIC index = FIC_pencillin_ + FIC_γ-mangostin_ = [Pencillin]/MIC_penicillin_ + [γ-mangostin]/MIC_γ-mangostin_; Synergy; <=0.5, No interaction; >0.5 - 4; Antagonism > 4.

## Discussion

Four crude extracts and five xanthones from pericarp of mangosteen inhibited growth of 5 serovars of *Leptospira* spp. with different efficacies. Various antimicrobials have also been reported to be active against a limited number of *Leptospira* spp. serovars [10]. The lowest MIC of all test xanthones against 5 leptospire serovars was 100 μg/ml which basically higher than the traditional antibiotics for the treatment of leptospirosis such as penicillin G (MIC_90_ = 1.56 μg/ml), amoxicillin (MIC_90_ = 3.13 μg/ml), ampicillin (MIC_90_ = 1.56 μg/ml), cefotaxime (MIC_90_ = 0.1 μg/ml), cefepime (MIC_90_ < 0.01 μg/ml), chloramphenicol (MIC_90_ = 6.25 μg/ml), doxycycline (MIC_90_ = 1.56 μg/ml), erythromycin (MIC_90_ < 0.01 μg/ml), and tetracycline (MIC_90_ = 1.56 μg/ml) [10]. Based on these results, it has been concluded that garcinone C and γ-mangostin belongs to 1,3,6,7-tetraoxygenetaed xanthones showed higher inhibitory activity. Similar findings were observed previously on 1,3,6,7-tetraoxygenetaed xanthones purified from mangosteen [41]. Increment of the alkyl groups in the xanthone nucleus of the 1,3,6-trihydroxylated series such as α-mangostin and garcinone D (Figure 1) reduced the antileptospiral activity.

In order to broaden the antileptospiral spectrum of xanthones, γ-mangostin was selected to test synergistic effect with penicillin G because of its low MIC and high abundance. The combination of this second major constituent γ-mangostin with penicillin G enhanced antileptospiral efficacy shown by a decrease in the MIC of both compounds, 4 to ≥500 times reduction of MIC for γ-mangostin whereas 2 to ≥40 times for penicillin G. An exception was observed for serovar Saigon in which the MIC of the combination was higher than that of penicillin G alone. The FIC index indicated the antileptospiral potential of the combination as no interaction for serovar Patoc, synergy for serovars Autumnalis, Bataviae, and Javanica, and antagonism for serovar Saigon. The mechanism of the synergistic effect is still unknown. But the role of penicillin is inhibition of peptidoglycan formation by binding to transpeptidases [42,43]. For γ-mangostin, it may work synergy with penicillin G in breakdown of bacterial membrane.

Mangosteen extracts have been used by the people in Southeast Asian countries as traditional medicine for treatment of several diseases such as abdominal pain, diarrhea, dysentery, infected wound, suppuration, and chronic ulcers without report of toxicity. The demonstrated antimicrobial activity suggest that xathones from mangosteen may be used as an alternative drug for the treatment of leptospirosis. Combination of γ-mangostin with penicillin G enhance antileptospiral efficacy resulting in the reduction of antibiotic consumption which may give a benefit to persons who develop allergy and side effects such as diarrhoea, hypersensitivity, nausea, rash, neurotoxicity, urticaria, and superinfection.

To date, γ-mangostin have been reported to induce apoptosis in human colon cancer cells [44] and has antagonistic effects which can be used in the treatment of inflammation, pain, and neuropsychiatric symptoms [45]. Mangosteen juice can promote health but need to be consumed together with fat-containing meal because the xanthones in mangosteen juice are absorbed when ingested along with a high-fat food [46]. The results of this study broaden the usefulness of xanthone from mangosteen in treatment of leptospirosis.

## Conclusions

The garcinone C and γ-mangostin from fruit of *G*. *mangostana* were found to be active against pathogenic leptospires but the MIC values were higher than those of antibiotics. The combination of γ-mangostin with penicillin G generated synergistic effect which enhanced efficacy for the treatment of leptospirosis.

## Competing interests

The authors declare that they have no competing interests.

## Authors’ contributions

WSE participated in study design and performed susceptibility and synergy testing. AJ isolated and purified xanthones from mangosteen. SS and PR provided mangosteen extracts and xanthone standards. CM designed bacterial susceptibility testing and revised manuscript. WS participated in study design, concluded the results, prepared and revised manuscript. All authors read and approved the final manuscript.

## Pre-publication history

The pre-publication history for this paper can be accessed here:

http://www.biomedcentral.com/1472-6882/13/182/prepub
